# Kidney disease and surveillance testing in children with Bardet–Biedl syndrome: an administrative data study

**DOI:** 10.1007/s00467-026-07470-9

**Published:** 2026-07-15

**Authors:** Gal Finer, Sarah E. Ward, Jeremy Pomeroy, Erica Davis, Sriram Ramgopal

**Affiliations:** 1 Division of Nephrology, Department of Pediatrics, Ann & Robert H. Lurie Children’s Hospital of Chicago, Chicago, IL USA; 2 Northwestern University Feinberg School of Medicine, Chicago, IL USA; 3 Fritz Wenzel Center for Clinical Research, Marshfield Clinic Research Institute, Marshfield, WI USA; 4 Stanley Manne Children’s Research Institute, Ann & Robert H. Lurie Children’s Hospital of Chicago, Chicago, IL USA; 5 Department of Pediatrics and Department of Cell and Developmental Biology, Feinberg School of Medicine, Northwestern University, Chicago, IL USA; 6 Division of Emergency Medicine, Department of Pediatrics, Ann & Robert H. Lurie Children’s Hospital of Chicago, Northwestern University Feinberg School of Medicine, Chicago, IL USA

**Keywords:** Bardet–Biedl syndrome, Chronic kidney disease, Administrative data, Pediatric health information system

Bardet–Biedl syndrome (BBS) is a multisystem ciliopathy in which kidney disease is a major determinant of morbidity and mortality [1, 2]. Multicenter pediatric data describing chronic kidney disease (CKD) and related surveillance in the USA are limited [1, 2]. The introduction of a new BBS-specific International Classification of Disease, Tenth Revision, Clinical Modification (ICD-10-CM) diagnosis code (Q87.83) in March 2023 provided an opportunity to identify affected children across large administrative datasets and describe coded kidney disease burden, kidney-relevant comorbidities, and documentation of surveillance testing.

We performed a multicenter retrospective cohort study using the Pediatric Health Information System (PHIS), identifying children younger than 18 years with an ICD-10-CM code for BBS documented between March 1, 2023, and June 30, 2025. After cohort identification, we evaluated all available inpatient and outpatient PHIS encounters for included children, including encounters preceding March 1, 2023, back to each child’s earliest PHIS encounter, to ascertain coded kidney disease, comorbidities, and CKD surveillance testing. To contextualize findings among children with BBS-associated CKD, we compared them with an age- and sex-matched cohort of children with CKD attributed to structural kidney anomalies.

We identified 294 children with BBS across 44 pediatric centers. Among the full BBS cohort, CKD was documented in 75 children (26%), and stage 5 CKD was documented in 18 (6%) (Table 1). Median age at first documented CKD was 5.9 years (IQR 2.5–10.5 years), and median age at first documented stage 5 CKD was 11.8 years (IQR 8.8–13.5 years).

**Table 1:** Coded kidney disease characteristics, comorbidities, and kidney surveillance testing among children with Bardet–Biedl syndrome (BBS) overall, children with BBS-associated chronic kidney disease (CKD), and age- and sex-matched children with structural anomaly-related CKD. Cells indicate *n* (%)

**Full BBS cohort**			
	***n* (%), *n* = 294**		
CKD, any stage	75 (26)		
Stage 5 CKD	18 (6)		
Kidney ultrasound	162 (55)		
Urine protein	62 (21)		
Urine creatinine	58 (20)		
**Matched CKD comparison**			
	**BBS–CKD**	**Structural Anomalies–CKD**	**OR (95% CI)**
	***n* (%), *n* = 74**	***n* (%), *n* = 296**	
0–2 years	11 (15)	44 (15)	-
3–17 years	63 (85)	252 (85)	-
Male	40 (54)	160 (54)	-
Female	34 (46)	136 (46)	-
Renal cysts	40 (54)	96 (32)	2.4 (1.5–4.1)
Obesity	53 (72)	41 (14)	15.7 (8.7–29.2)
Obstructive sleep apnea	24 (32)	49 (17)	2.4 (1.4–4.3)
Type 2 diabetes	5 (7)	8 (3)	2.6 (0.8–8.1)
Hypertension	23 (31)	105 (35)	0.8 (0.5–1.4)

Kidney surveillance testing in the full BBS cohort was variably documented within PHIS. Kidney ultrasound was documented in 162 children (55%), urine protein testing in 62 (21%), and urine creatinine testing in 58 (20%). Among children with BBS-associated CKD included in the matched comparison, renal cysts were more commonly coded than in children with structural anomaly-related CKD (54% vs. 32%; OR, 2.4; 95% CI, 1.5–4.1). Obesity and obstructive sleep apnea were also more frequent in the BBS–CKD cohort (72% vs. 14%; OR, 15.7; 95% CI, 8.7–29.2; and 32% vs. 17%; OR, 2.4; 95% CI, 1.4–4.3, respectively). Type 2 diabetes was numerically more frequent in the BBS–CKD cohort, although event counts were small and the association was not statistically significant, whereas hypertension was not clearly different between groups (Table 1). The higher burden of metabolic comorbidities observed in the BBS-associated CKD cohort is consistent with emerging evidence of the multifactorial nature of BBS–CKD [3] and supports consideration of more standardized kidney surveillance within multidisciplinary BBS care [4].

This study should be interpreted in the context of administrative data limitations. PHIS captures encounters within participating pediatric hospitals and may underrepresent children followed in non-PHIS settings. Genotype, laboratory values, anthropometrics, and imaging performed outside participating hospitals are not available. Therefore, these findings should not be viewed as defining the full renal phenotype of BBS, which is heterogeneous and influenced by both genetic and nongenetic contributors [3, 5]. Rather, these data provide a multicenter US snapshot of coded kidney disease burden, comorbidities, and CKD-related surveillance testing among children identified using the BBS-specific ICD-10-CM code and support consideration of more standardized CKD surveillance [3, 4].

## Data Availability

The data that support the findings of this study are not publicly available because they were obtained from the Pediatric Health Information System (PHIS), which is owned by the Children's Hospital Association and subject to datause restrictions. Researchers who meet the criteria for access may obtain the data directly from the Children's Hospital Association (https://www.childrenshospitals.org/content/analytics/product-program/pediatric-health-information-system).
